# Anterior cruciate ligament surgery in the rabbit

**DOI:** 10.1186/1749-799X-8-27

**Published:** 2013-08-19

**Authors:** Manon Bachy, Ines Sherifi, Fréderic Zadegan, David Petrover, Hervé Petite, Didier Hannouche

**Affiliations:** 1 Laboratoire de Bioingénierie et Bioimagerie Ostéo-Articulaire (B2OA) CNRS UMR 7052, Université Denis Diderot Paris VII, 10 avenue de Verdun, Paris 75010, France; 2 Department of Orthopaedic Surgery, Hôpital Lariboisière, AP-HP, Paris 75010, France

**Keywords:** Anterior cruciate ligament, Allograft replacement, Rabbit, Cell survival, Ligamentization

## Abstract

**Background:**

Various methods regarding allograft knee replacements have been described. The animal models, which are generally used for this purpose include sheep, dogs, goats, and pigs, and accrue significant costs for study protocols. The authors herein describe an efficient and cost-effective model to study either native or tissue-engineered allografts for anterior cruciate ligament (ACL) replacement in a New Zealand rabbit model with the potential for transgenic and cell migration studies.

**Methods:**

ACL reconstructions were performed in rabbits under general anesthesia. For fresh allograft implantations, two animals were operated in parallel. Each right extensor digitorum longus tendon was harvested and prepared for implantation. After excision of the ACL, tibial and femoral bone tunnels were created to implant each graft in the native ACL position.

**Results:**

During a 2-year period, the authors have successfully undertaken this surgery in 61 rabbits and have not noticed any major complications attributed to this surgical technique. In addition, the authors have observed fast recovery in the animals postoperatively.

**Conclusion:**

The authors recommend this surgical procedure as an excellent model for the study of knee surgery.

## Introduction

Tears of the anterior cruciate ligament (ACL) are among the most common ligamentous injuries with more than 200,000 patients being diagnosed yearly in the USA alone [1,2]. Over the past decades, the management of ACL ruptures has substantially evolved from non-operative treatment to ACL reconstruction using tendon grafts, due to considerable improvements made in the field of knee arthroscopy and tendon graft fixation. The long-term success rates of ACL replacements depend on the healing and osteointegration of the soft tissue graft in the bone tunnel; this aspect is essential to achieve knee stability and to allow patients to return to sport activities. Despite advances in surgical techniques and patient treatment, important challenges remain regarding the healing process of the ACL replacement grafts. Potential means to accelerate and promote tendon graft incorporation can be developed either with the use of growth factors or stem cell-based therapies.

This important clinical challenge has promoted research using numerous animal models including goats [3], sheep [4], rabbits [5], Yucatan minipigs [6], and dogs [7]. The wealth of translational research on this topic aims at developing optimal animal models that closely mimic the condition in humans and would thus help define, develop, and compare new treatment strategies for successful and efficient replacement of the ACL. In particular, tissue engineering approaches, which aim at designing and building living tissues for patients in the laboratory, have shown great potential in providing cell-containing constructs for future ACL replacement; such alternatives have the potential for overcoming the drawbacks associated with synthetic allografts.

The ACL replacement technique in an animal model poses technical difficulties for researchers. Many researchers thus choose to conduct animal studies using larger animal models such as goat, sheep, and dog. Because these larger animals can be difficult to maintain and require more complex procedures (such as specific equipment, additional caregivers, and anesthesia care), we chose to perform this surgery in rabbits, which are easier to handle and require little technical assistance. Rabbits are small animals and, compared to larger animals, generally require simpler housing and care protocols. Also, recent work has focused on improving rabbit welfare, treating pain and minimizing their stress. In addition, the genetic makeup and health conditions of rabbits are well studied; transgenic models are currently used in research, thus explaining the wide use of the rabbit as an animal model. The use of the novel surgical technique in the rabbit avails researchers with a more cost-effective animal model as well as opens up the opportunity of using transgenic rabbits in order to monitor cell migration and survival and to evaluate graft incorporation in the recipient animals. Our team is currently using a transgenic rabbit model that allows assessment of cell migration in ACL allografts. In the present report, we present our experience with this technique for studying allograft implantation in a versatile and cost-effective animal model.

## Methods

### Rabbits

Sixty-one male New Zealand white rabbits (Crl:KBL(NZW)) were obtained from Charles River Laboratories (Lieu-dit Oncins, France). The strain origin of these animals was Charles River Canada in 1991 from the Kitayama Breeding Laboratories (Japan). At the time of the present study, the animals on arrival were approximately 3 months old and each one weighed between 2 and 2.5 kg. Microbiology tests at that time established that these rabbits were pathogen free (SPF) and virus antibody free (VAF/Plus®, Charles River Laboratories, Inc., Raleigh, NC, USA). After land transport, the animals underwent an acclimatization period of at least 48 h before experimentation. For surgery and postoperative maintenance, the animals were cared for in accordance with the directives of the Animal Experiment Ethics Committee of Lariboisiere-Villemin (number 09, CEEA-LV/2010-01-01). During the experiments, a closed system (A1 conventional animal facilities; CNRS, France) was maintained to protect the animal microbiological status. These rabbits were housed in individual, unbedded, stainless steel cages (each 64 × 54 × 40 cm^3^) allowing visual, hearing, and olfactory contact, and thus minimizing stress during the present study. The animal cages were cleaned three times a week in accordance with optimal cage cleaning recommendations. All rabbits were exposed to a 12:12-h light/dark cycle. The animal facility room temperature was maintained at 17°C to 21°C and the relative humidity between 50% and 55%. All rabbits had *ad libitum* access to water distributed by an automatic watering system (whose valves were tested once daily for patency) and were fed a commercially available food diet (high-fiber diet Altromin 2120/2123, Genestil, Royaucourt, France) daily. The animal studies reported here were performed between January 2009 and June 2010.

### Personnel and materials for the surgical procedure

Three people (one researcher, one animal surgeon, and one technician) conducted the procedures described in the present study. One team member prepared the animals before surgery in the induction room, administered anesthesia, and handed the sterile surgical equipment to the other members of the team during surgery. Two team members in the sterile area prepared all materials needed for and conducted the surgical procedure: one member prepared the allograft tendon while the other prepared the surgical site. All personnel used appropriate protective equipment in accordance with the regulations of the institute where the study was performed.

The following materials were needed for surgery: sterile gauze, alcohol-free iodine solution, syringes (1 and 5 mL), needles (25 and 18 G), sterile gowns, surgical caps, surgical masks, sterile gloves, sterile drapes, shaver, sterile stockings, surgical blades (numbers 23 and 11), ophthalmic blades, sterile surgical boxes (two pickups, scissors, and needle driver), sterile cups, 2.0-mm drill bit and drill motor, Prolene 3.0 sutures (Ethicon Inc., Somerville, NJ, USA), Ethilon 3.0 sutures (Ethicon Inc.), Vicryl 3.0 sutures (Ethicon Inc.), elastic bandages, and sterile phosphate-buffered saline (PBS).

### Anesthesia and preoperative preparation

The ACL reconstruction procedure was performed in the rabbits under general anesthesia administered gradually by intramuscular injections of anxiolytic, muscle relaxant, and sedative drugs; this approach alleviated the stress of intravenous anesthesia administration to the animals. The rabbits were anesthetized through an intramuscular (IM) injection of anxiolytic (diazepam (0.5 mg/kg)), muscle relaxant (medetomidine hydrochloride (290 μg/kg)), and sedative (ketamine 500 (50 mg/kg)) drugs to ensure an appropriate balance of deep sedation while maintaining spontaneous ventilation and avoiding morbidity associated with intubation (including traumatic intubation, traumatic extubation, pneumothorax, or pneumonia) during an average surgical procedure time period of 40 min. Animal anesthesia was induced in a calm, quiet, and non-stressful environment. IM injection was administered at the lateral muscular compartment of the thigh of the contralateral (to the operated) leg. For the purpose of antibiotic prophylaxis, rilexine (0.16 mL/kg) was administered by a single intramuscular shot prior to skin incision. Sterile drapes were used for optimal sterility of the surgical field, while exposing the thoraces of the animals in order to allow direct visualization of the animal respiratory rate. The right knee of each rabbit was shaved anteriorly and prepped using alcohol-free iodine solution.

### Surgical technique

**Figure 1:**
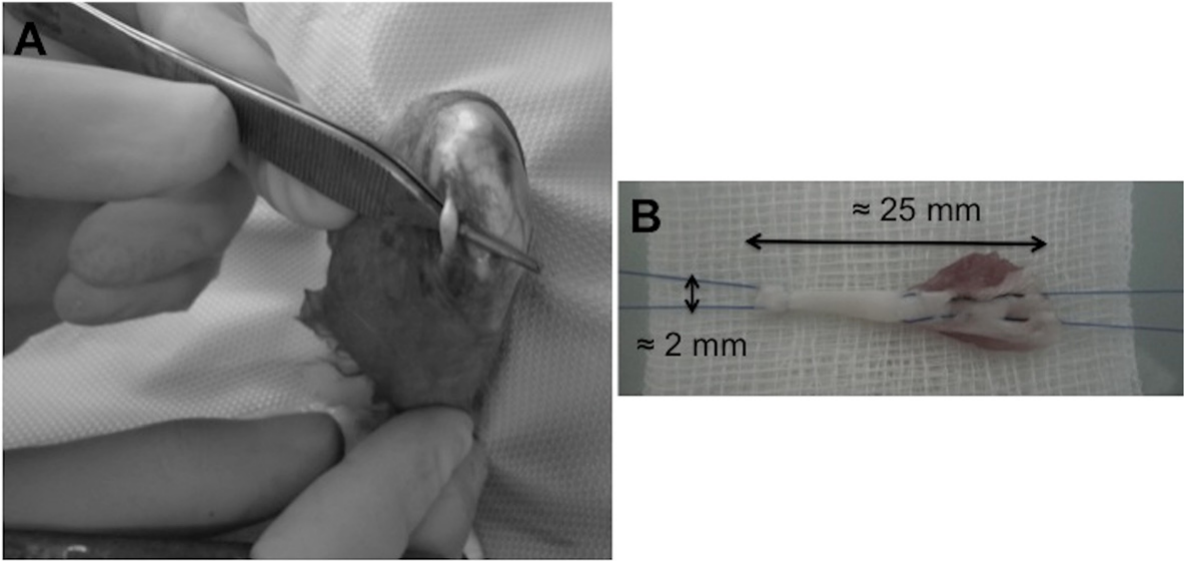
**EDL tendon harvest and allograft preparation. (A)** The EDL tendon is located close and lateral to the patellar tendon. **(B)** A minimum length of 25 mm is preferred. Diameter is approximately 2 mm. A Kessler suture is performed at the proximal end and a whip stitch suture at the other end.

**Figure 2:**
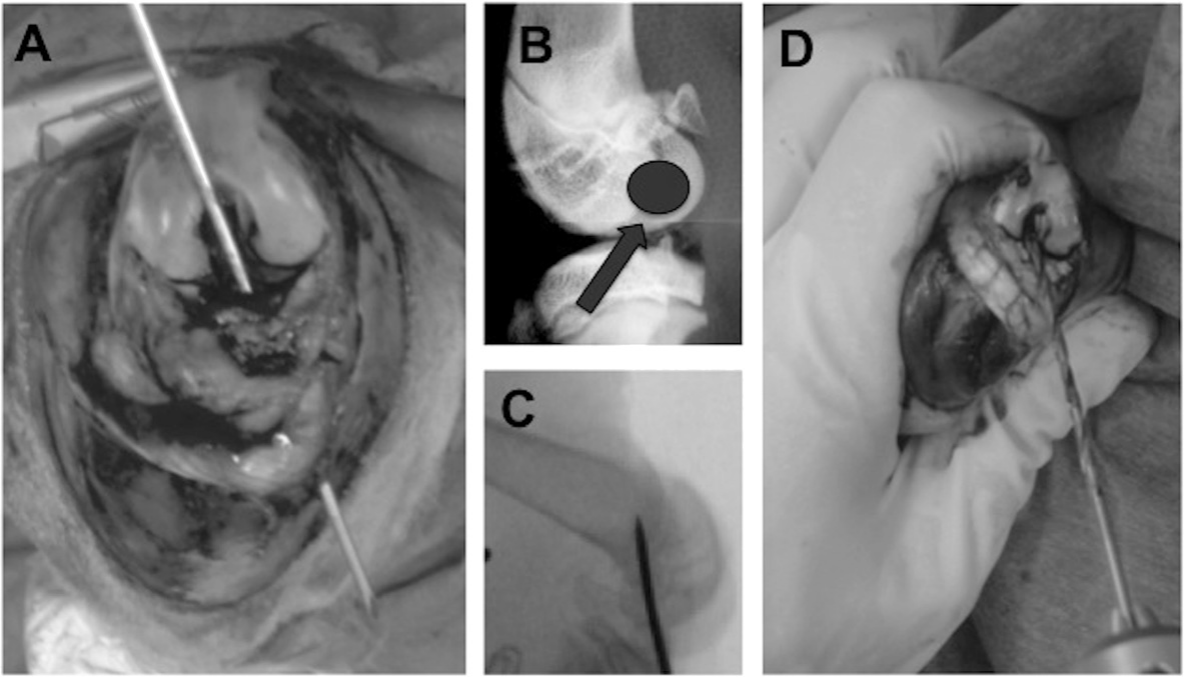
**Preparation of the tunnels. (A)** After excision of the ACL, the tibial bone tunnel was created using anatomical insertions of the ACL. **(B)** Particular attention was given to the femoral bone tunnel in order to achieve isometric placement of the graft. **(C)** Correct posterior placement of the femoral bone tunnel was ensured using intraoperative X-ray. **(D)** 2.0-mm bone tunnels were drilled. Please note the small size of the rabbit knee compared to the size of the surgeon's fingers.

**Figure 3:**
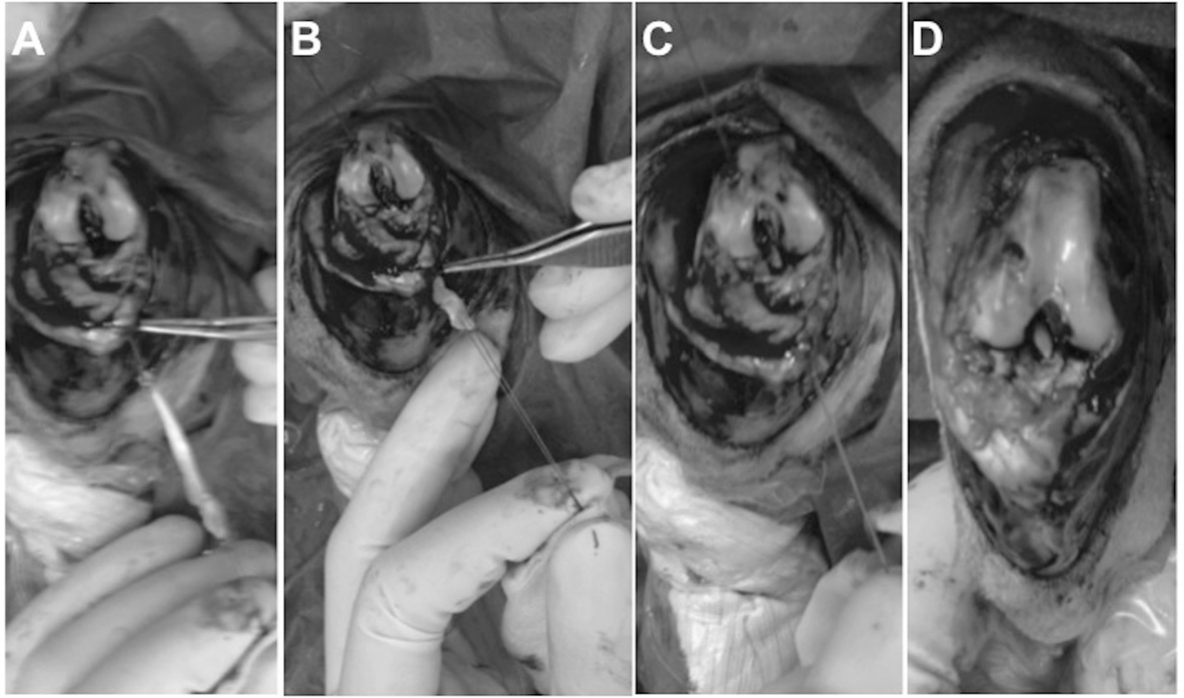
**Allograft fixation. (A)** Allograft preparation. **(B)** Fresh, hydrated allograft was passed through the tibial tunnel first. **(C)** Application of tension before fixation. **(D)** Suturing to the periosteum and final views of the procedure.

ACL reconstruction was performed under sterile conditions. Each rabbit was positioned in the supine position and covered with a sterile drape exposing only the right knee area. The foot was encased in an orthopedic stocking for sterility purposes. A median knee incision was performed and the extensori digitorum longi (EDL) tendon was harvested (Figure 1A). From that time until implantation, the EDL graft was kept immersed in PBS at room temperature for approximately 15 min. With the rabbit leg in extension, a medial para-patellar arthrotomy was performed, and the patella was dislocated laterally. This leg was placed in extreme flexion to expose the ACL. After excision of the ACL, tibial and femoral bone tunnels were created to implant the graft at the ACL native position. To create the bone tunnels, holes (each 2.0 mm in diameter) were drilled at the anatomical insertions of the ACL for optimal isometry of the ACL reconstruction (isometric angle of the ACL reconstruction). Reproducible and accurate placement of the drill holes in relation to the original ACL insertion sites was necessary to achieve loading conditions similar to those of the native ACL. Particular attention was given to the femoral bone tunnel. While one of the surgery team members was drilling the bone tunnels (Figure 2), another member was preparing the allograft (Figure 1B). The fresh, hydrated allograft was prepared using whip stitch suture and, then, was passed through the tunnels (Figure 3). The allograft ends under tension were sutured to the periosteum with the knee in extension using non-absorbable 3.0 Prolene sutures. The incision was closed in layers: arthrotomy and subcutaneous layers using Vicryl sutures and the skin using Ethilon sutures. Sterile gauze was used for dressing. Postsurgery, the knee was maintained in extension using elastic bandage for stability and pain control. The joint immobilization dressing was left in place until each rabbit gave the first sign that it was no longer needed by starting to remove it on its own.

### Postoperative animal care and specimen collection

The husbandry and housing methods followed in the present study were previously described. Analgesia was performed by systematic administration of meloxicam (0.02 mg/kg) for 24 h. The operated knee was immobilized in extension using an elastic bandage for a period of 5 days postsurgery. All rabbits were allowed to move freely and resumed normal activity 2 days after the surgery. General examination of these animals was performed daily to detect any clinical sign of pain and other complications such as anorexia, abnormal cry, decreased activity, and leg dressing problems.

For specimen collection, general anesthesia was induced to each rabbit as previously described at 3 and 6 weeks. The rabbits were sacrificed while fully anesthetized, using intracardiac injection of approximately 5 mL of a mixture (containing 1 g of pentobarbital sodium, 52 mg of benzyl alcohol, and 0.05 mg of Ponceau 4R (E124). Cessation of respiration, absence of heart sounds, and absence of reflexes were checked to confirm the death of each rabbit.

### Imaging

A 64-slice arthro-computed tomography (CT) (General Electric, Fairfield, CT, USA) was used to image the allograft in the knee joint of the first three operated rabbits. Resolution was 0.3 × 0.6 mm^2^. Injection of iodine contrast within both operated knee cavities and the contralateral non-operated knee was used to confirm proper initial placement of the graft with respect to the native ACL. Imaging was performed within 2 h following rabbit euthanasia.

## Results

### Surgical procedure

There were no animal deaths due to the administered anesthesia. In addition, neither respiratory nor cardiac abnormalities were observed in the rabbits after anesthesia. All medication dosages described in the ‘Methods’ section were appropriate for the animal size, weight, and type; the respective medication effect lasted for approximately 40 min. There were no early awakenings of the animals during the surgical procedure. The entire surgical procedure lasted approximately 40 min; this time period provided ample time for induction of anesthesia, surgery, dressing of the wound, and subsequent transferring of the rabbits to their individual cages.

### Postoperative care

All rabbits tolerated the surgery well and exhibited no signs of postoperative infection. The animals returned to normal activity in their cages 2 days after surgery. No signs of pain, wound infection, or abnormal behavior were detected for the duration of the study. Assessment and localization of the incision site was easily done in the shaved leg area. None of the rabbits required either a second dressing or prolonged immobilization. After an initial weight loss period of approximately 5 days postsurgery, the weight curve was normalized, and at 3 weeks, all rabbits surpassed their respective preoperative weight. At 6 weeks, the average increase in body weight averaged 550 g, and the gain in body weight was approximately 19%. The rabbits had a noticeable limp up to 3–4 weeks after surgery but recovered well thereafter and had a symmetrical jump.

### Imaging

**Figure 4:**
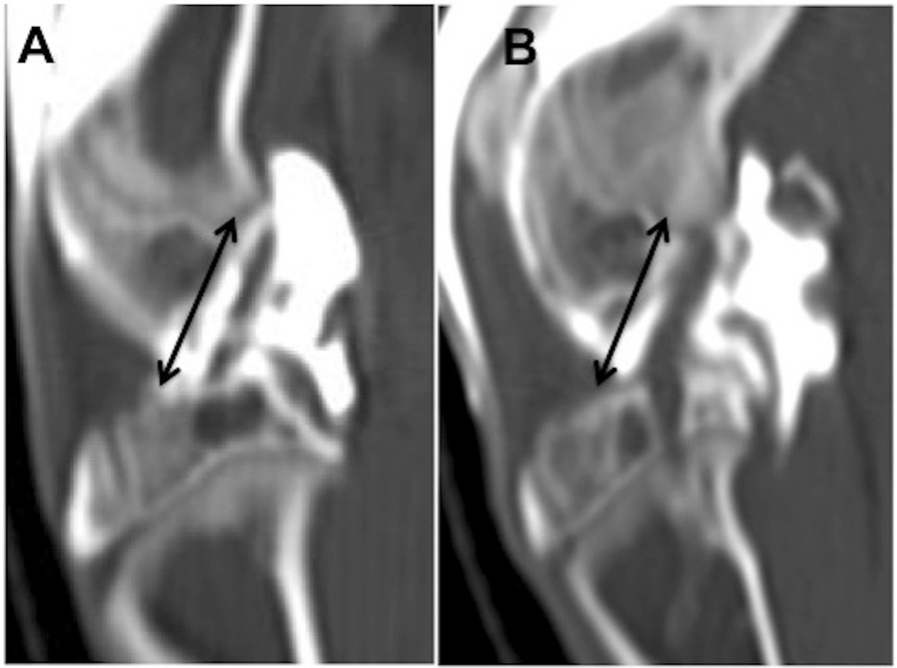
**Representative arthro-CT scan for graft placement evaluation. (A)** Injection of contrast material into the knee joint revealed the axis of the native ACL. **(B)** Operated knee exhibiting the same orientation.

Arthro-CT imaging analysis provided evidence regarding proper surgical insertion of the graft in the knee joint. The orientation of implanted grafts with respect to the axes of the tibia and femur was similar to that of the native ACL in sagittal cross-section (Figure 4).

### Macroscopic dissection

**Figure 5:**
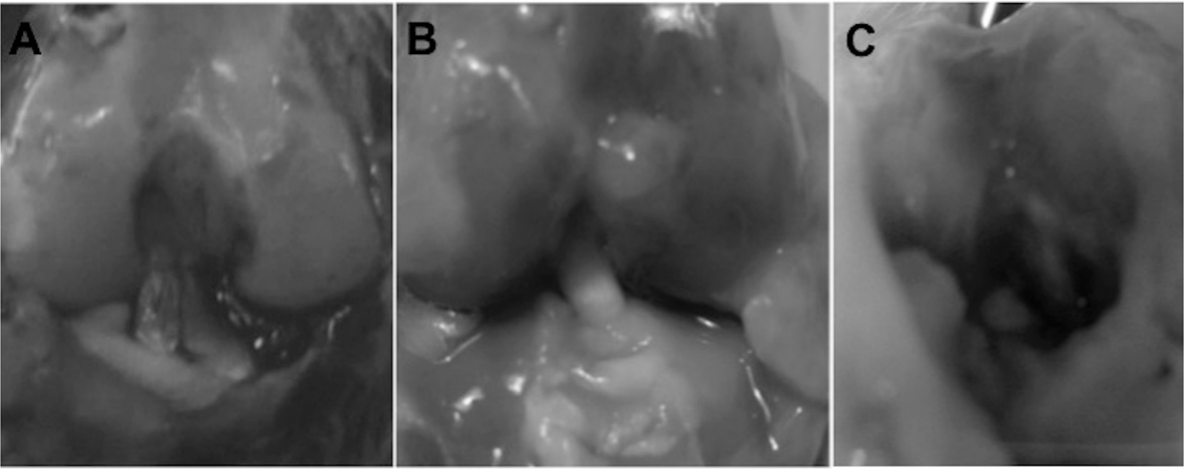
**Macroscopic views after pullout testing. (A)** Native ACL. **(B)** Good aspect of a graft at 3 weeks postoperatively. **(C)** Case of rupture of an ACL graft at 6 weeks.

At 3 and 6 weeks postoperatively, dissection of the knee assessed the quality and the integration of the implanted grafts. We used macroscopic and pullout tests at the time of sacrifice at 3 and 6 weeks postsurgery. This analysis provided information regarding cases of graft integration (Figure 5A,B) and rupture (Figure 5C).

## Discussion

The technique performed in this paper closely mimics ACL repair surgeries in humans. The New Zealand white rabbit provides an excellent cost-effective animal model for studying ACL surgery [8]. Because the rabbits are smaller, animal maintenance and housing is simpler compared to the procurement and upkeep of large animal such as sheep. In addition, it has been our experience that the rabbits recover very well postoperatively, often resuming mobility within hours after surgery. All rabbits in the present study regained full preoperative activity and symmetrical jumps 3 weeks postsurgery [9].

The small-animal model described herein is valuable to surgeons and researchers working on ACL repair as it describes an easy, safe, and reproducible model of ligamentoplasty, which can be used to improve our knowledge regarding tendon graft integration as well as to test new treatment strategies such as stem cell or growth factor therapy; the aim is not necessarily to replicate human disease conditions but to compare different treatment modalities in the same animal model. Importantly, as compared to large-animal models, the rabbit has noticeable similarities. The caudal tibial slope of all quadrupeds commonly used to study ACL reconstruction is steeper than in humans, which must be kept in mind when considering potential applications of these studies in humans [10]. In contrast to those of other quadruped mammals, the only animal whose knee joint displays an extended resting position, as in humans, is the elephant, which cannot be considered for experimental purposes [11].

The surgical and anesthesia procedures described in the present study were performed successfully without the need for intubation and ventilation. The rabbits remained adequately sedated during a technically challenging procedure; their respiratory status was preserved, and thus, complications associated with intubation and mechanical ventilation (including, but not limited to, intubation difficulty, traumatic intubation or extubation, pneumothorax, and pneumonia) were avoided. With the current emphasis on cost containment and efficiency, the method described in this paper has the advantage that it can be single-handedly conducted by one person prior to surgery. An important drawback of the method is that it requires three intramuscular injections (specifically, anxiolytic, relaxant, and sedative) to maintain the animals comfortable during the surgical procedure.

From a surgical viewpoint, the EDL tendon is often chosen as a graft [12,13] since, lateral to the patella, it is easily accessible and of sufficient length to replace the ACL. It is helpful to harvest the longest possible segment of EDL tendon in order to ensure a long enough allograft for the length of the knee joint, crossing of the bone tunnels, and for suturing; for these reasons, the preferred minimum length is 2.5 cm. Rabbit morbidity is acceptably low because rabbits do not use the EDL for jumping [14]. In the present study, the EDL was fixed in the joint in the same axis as the ACL. For best results, it is important to obtain extreme flexion (when drilling the bone tunnels) and extension (when suturing the graft to the periosteum under tension) of the knee. Initially, the correct posterior placement of the bone tunnel in the femur was ensured by intraoperative X-ray (Figure 2B). Graft positioning was validated using arthro-CT scanning in order to compare the implanted graft to the native ACL axis in the contralateral leg. The endpoints of each graft were sutured to the periosteum to leave the bone tunnels free of material (for histological purposes) and to allow proper integration of the graft [15-18]. Non-absorbable sutures were used so that tissue healing could proceed unaffected. A suture over a button technique was not chosen because of the reported increased infection rate around such suture sites [19]. All rabbits tested were approximately 3 months old. At that age, their epiphyseal plate is still open. We do not anticipate that animal growth will affect the range of motion of the operated knee. A possible complication that may occur with rabbit aging is partial epiphysiodesis since the bone tunnel will change with epiphyseal growth; the short follow-up of the present study, however, did not allow evaluation of this aspect which needs to be addressed in future studies.

The objective of the present study was not to compare the long-term effect in ACL-deficient or ACL-reconstructed knees, which would have required longer endpoints and a negative control group with transected ACL; instead, we aimed at and were successful in developing an ACL reconstruction procedure with a safe anesthesia and a fast, safe, and reproducible surgical technique that allows further studies on osteointegration in the bone tunnels and the ligamentization process. We believe that the anesthesia and surgical techniques described here minimize morbidity during ACL surgery and provide a fast, safe, and reproducible animal model for the study of ligament healing. In addition, transgenic rabbits are available and constitute an important resource for expanding the scope of research to address cell migration and new disease models *in vivo*[20,21]. Availability of genetically distinct animals provides a unique opportunity for the study of graft integration and healing. For instance, since rabbit antibodies are readily available, the model established in the present study provides a unique opportunity for immunohistochemistry and protein marker studies.

In conclusion, the New Zealand white rabbit provides an excellent model for studying ACL surgery. Future improvements to this *in vivo* model may include incorporation of either synthetic matrices or tissue-engineered constructs in place of the EDL tendon in order to ultimately produce alternative, successful, efficient, and durable ACL replacement methodologies.

## Competing interests

The authors declare that they have no competing interests.

## Authors’ contributions

MB made substantial contributions to the conception, design, and acquisition of data. IS drafted the manuscript and critically revised it for important intellectual content; she also assisted in the acquisition of data. FZ participated in the design of the study and assisted in the animal surgery. DP conducted the imaging analysis and participated in the interpretation of the data. HP contributed to the design of the study and critically revised the final manuscript. DH conceived the study and participated in its design and coordination. All authors read and approved the final manuscript.
